# How strong is the association between abdominal obesity and the incidence of type 2 diabetes?

**DOI:** 10.1111/j.1742-1241.2008.01805.x

**Published:** 2008-09

**Authors:** N Freemantle, J Holmes, A Hockey, S Kumar

**Affiliations:** 1 School of Primary Care, Occupational and Public Health, University of BirminghamBirmingham, UK; 2 PMSI HealthcareLondon, UK; 3 sanofi-aventis, Guildford SurreyUK; 4 Clinical Sciences Research InstituteUniversity of Warwick, Warwick, UK

## Abstract

**Background:**

Quantitative evidence on the strength of the association between abdominal obesity and the incidence of type 2 diabetes was assessed.

**Methods:**

Systematic review of longitudinal studies assessing the relationship between measures reflecting abdominal obesity and the incidence of type 2 diabetes.

**Results:**

There was a strong association between measures reflecting abdominal obesity and the incidence of type 2 diabetes, the pooled odds ratio was 2.14 (95% CI: 1.70–2.71; p < 0.0001). Waist circumference (WC) was at least as good as other measures in predicting outcome.

**Conclusions:**

There is a strong association between measures reflecting abdominal obesity and the development of type 2 diabetes. Reducing WC may reduce the risk of developing type 2 diabetes.

**Review Criteria**

- Comprehensive searches of Medline and Embase undertaken in March 2006. Exclusion criteria agreed by authors.
- Studies included in the review if they examined the relationship between at least one measure of abdominal obesity and development of type 2 diabetes over time.
- Abstracts of all identified papers assessed by two reviewers. Inter-rater agreement for study selection measured using kappa statistic.
- Data from 10 longitudinal studies included in the quantitative analysis.

**Message for the Clinic**

- On average, raised abdominal obesity increases risk of type 2 diabetes more than twofold.
- All measures used to capture abdominal obesity show a strong relationship to the incidence of type 2 diabetes.
- Clinicians can use a simple measure of abdominal obesity to identify patients at increased risk of developing type 2 diabetes.
- Effective targeting of new drug therapies towards those at higher risk may be greatly improved by systematic measurement of waist circumference.

## Introduction

The prevention and treatment of diabetes is a public health concern in many health systems. There is a substantial literature referring to obesity as a major risk factor in the development of diabetes. These studies have used body mass index (BMI) as the measure of obesity. It is however increasingly recognised that for a given BMI, central rather than lower body fat distribution, confers greater risk of metabolic and cardiovascular complications of obesity (1). Schmidt et al. (2) cite studies dating back to 1956 indicating the importance of the association between waist–hip ratio (WHR) and type 2 diabetes.

The objective of this review was to assess the quantitative evidence on the relationship between abdominal obesity and the incidence of type 2 diabetes in both men and women, and to examine the relative usefulness of different measures of abdominal obesity.

## Method

Comprehensive searches of Medline and Embase were undertaken by the authors in March 2006, including an extensive list of subject area key terms. Studies of human subjects published in English since 1985 were considered. Exclusion criteria were studies dealing with HIV, hormonal treatment, vitamins or transplantation, and studies in patients with comorbidities at baseline. A total of 119 papers were identified and screened for relevance by title and abstract. A subset of 20 relevant papers were then included in the review.

Studies were included in the review where they examined the relationship between at least one measure of abdominal obesity and the development of type 2 diabetes over time. Measures of abdominal obesity considered in the review were waist circumference (WC), WHR, iliac circumference (IC) and intra-abdominal fat area (IAFA). BMI was not considered a measure of abdominal obesity.

Abstracts of all the identified papers were assessed by two reviewers. Inter-rater agreement for study selection was measured using the kappa statistic. The weighted kappa was 76.8%, showing a good level of agreement, and inclusion of the balance of papers was agreed through discussion on the basis of the full papers. Hand searching identified two further papers.

Data from 10 longitudinal studies reporting the relation of WHR, WC, IC or IAFA to the development of type 2 diabetes on a ratio scale [using odds ratios (OR) or relative risks] were then included in the quantitative analysis.

### Statistical analysis

We constructed a mixed model to pool the reported log ratio outcome (e.g. log OR or log-relative risk) from a total of 15 cohorts reported in these studies. As studies all estimated the relationship between measures of abdominal obesity and the development of diabetes in different ways, we did not attempt to pool a single fixed relationship but a distribution of effects and so a random effects analysis was prespecified. Thus, between study heterogeneity in the definition of metrics and adjustments for confounding performed was addressed through defining studies as random effects. We treated analyses of different measures of abdominal obesity within a cohort as repeated measures. Given large sample sizes and small event rates, the OR approximates closely the hazard ratio, but to avoid confounding by type of analysis, we adjusted for risk- or odds-based outcome. Studies were weighted in the analysis using the inverse of the within study variance. The principal analysis was to estimate the pooled effect of abdominal obesity and the development of diabetes, regardless of measurement method used. The relative effectiveness of WC and alternative methods of measurement, and the potential confounding effect of length of follow up were estimated directly from the model. All analyses were conducted in Proc Mixed, in the sas statistical program (SAS version 9.1, SAS Institute, Cary, NC).

## Results

### Studies included

Table 1 summarises the characteristics of the studies used for quantitative analysis. Eight studies (13 cohorts) used fasting and/or 2 h glucose tolerance tests to identify subjects with diabetes, three of which (four cohorts) also used treatment with diabetic medication as an alternative. Two studies used subject self-reporting, confirmed by random sampling of medical records. All 15 cohorts in the analysis were adjusted for age, eight were adjusted for BMI and most included a range of other adjustment factors.

**Table 1:** Characteristics of studies used for quantitative analysis

References	Sample size	Sample age (years)*	Sample gender	Sample ethnicity	Follow-up period	Abdominal obesity measure	Adjustment factors
**Diabetes diagnostic criteria: fasting glucose and glucose tolerance tests**							
Cassano et al. 1992 (3)	1972	Mean 41.9	Male	98% Caucasian	Mean 18 years	WHR	Age, BMI, smoking
Snijder et al. 2003 (4)	619	Mean 60.2	Male	Caucasian	6 years	WHR, WC	Age (hip circumference and BMI, thigh circumferenceand BMI adjusted results were also reported)
Snijder et al. 2003 (4)	738	Mean 60.4	Female	Caucasian	6 years	WHR, WC	
Wang et al. 2005 (5)	22,270	40–75	Male	USA	13 years	WHR, WC	Age, BMI, smoking, physical activity, alcoholconsumption, trans fat and cereal fibre intake
Wei et al. 1997 (6)	270	Mean 42.2	Male	Mexican Americans	Mean 7.2 years	WHR, WC	Age (results for other anthropometric variableswere also reported)
Wei et al. 1997 (6)	451	Mean 43.4	Female	Mexican Americans	Mean 7.2 years	WHR, WC	Age (results for other anthropometric variableswere also reported)
McNeely et al. 2001 (7)	466	Mean 52.2	Both	2nd generationJapanese Americans	5 years	WC	Age, sex, smoking, family history
Chihaoui et al. 2001 (11)	271	Mean 47.5 at follow-up	Male	Tunisian	10 years	IC	Age, BMI, BP, baseline glucose, insulin, cholesterol
Chihaoui et al. 2001 (11)	430	Mean 44.0 at follow-up	Female	Tunisian	10 years	IC	
**Diabetes diagnostic criteria: measured insulin and glucose concentration**							
Wang et al. 1997 (8)	995	Mean 54.2	Male	Chinese	Mean 3.3 years	WHR, WC	Age (insulin adjusted results were also reported)
Wang et al. 1997 (8)	1195	Mean 52.0	Female	Chinese	mean 3.3 years	WHR, WC	
**Diabetes diagnostic criteria: American Diabetes Association 1997 criteria†**							
Boyko et al. 2000 (12)	290	Mean 61.8	Both	2nd generationJapanese Americans	10 years	IAFA	Age, sex, IGT at baseline, family history, non-IAFA,fasting C-peptide, insulin response
Boyko et al. 2000 (12)	230	Mean 40.1	Both	3rd generationJapanese Americans	10 years	IAFA	
**Diabetes diagnostic criteria: self-reported diabetes or medical records**							
Kaye et al. 1991 (9)	41,837	55–69	Female	USA	2 years	WHR	Age, BMI, education
Carey et al. 1997 (10)	42,492	30–55	Female	USA	8 years	WHR	Age, BMI, family history, exercise, smoking,dietary intakes

* Age at baseline unless otherwise specified. †Taking oral hypoglycaemic medication or insulin, or fasting plasma glucose ≥ 7.0 mmol/l or 2-h value ≥ 11.1 mmol/l. WHR, waist–hip ratio; WC, waist circumference; IC, iliac circumference; IAFA, intra-abdominal fat area; BMI, body mass index; BP, blood pressure; IGT, impaired glucose tolerance.

### Waist and/or waist–hip ratio and incidence of diabetes

All studies showed a positive association between waist or WHR and incidence of diabetes. Cassano et al. (3) used a proportional hazards model based on a prospective evaluation of male participants in the Department of Veterans Affairs Normative Aging Study cohort. They found that, after adjusting for age, BMI and cigarette smoking, men in the top tertile for the ratio of abdominal circumference to hip breadth had a 2.4-fold greater risk of diabetes than did men in the lowest tertile (95% CI: 1.7–3.7). When blood glucose was analysed as a continuous outcome variable, the findings were consistent, i.e. there was a positive association with abdominal fat independent of total-body adiposity.

Snijder et al. (4) reported data from the Hoorn study indicating lower OR than Cassano et al., but with a higher OR in women than men for both WHR and WC. When adjusted for hip circumference and BMI or thigh circumference and BMI, the OR for WC were higher in both men and women, the highest being 2.66 per one SD larger waist for the latter adjustment in women.

Wang et al. (5) reported data from the US Health Professionals Follow-Up Study showing that WC was better than either BMI or WHR in predicting type 2 diabetes. 83.6% of type 2 diabetes was identified in the fifth decile of WC (compared with 82.5% for the fifth decile of BMI and 74.1% for the fifth decile of WHR). However, they point out that the influence of abdominal fatness on type 2 diabetes is a continuous one so any cut-offs are arbitrary.

In a study of Mexican Americans Wei et al. (6) found that WC was the best obesity-related predictor of non-insulin-dependent diabetes, with a predictive effect equal to that of WHR and BMI combined. The authors argue that abdominal localisation of body fat is a more important determinant than total amount of body fat in this population (mean age 42 for men, 43 for women).

In Japanese Americans McNeely et al. (7) found that in their younger subgroup aged ≤ 55 years (*n*= 240), a WC greater than or equal to the third tertile (> 91.5 cm for men, > 80.2 cm for women) was associated with diabetes (adjusted relative risk 5.4; 95% CI: 1.7–17.0). This was substantially higher than their overall findings for the > 55 and ≤ 55 age groups combined.

In Taiwan, Wang et al. (8) reported data showing a stronger relationship between diabetes incidence and obesity in women than in men, and indicating that WC was a better predictor than WHR. However, for women higher standardised relative risks were reported for subscapular skinfold thickness (3.07) and BMI (2.79).

Kaye et al. (9) used participant self-reporting of diabetes and found that WHR was a significant independent predictor of diabetes in a dose–response fashion in older women. In addition, women in the highest tertiles of both WHR (> 0.878) and BMI (> 29.2 kg/m^2^) had a 14.4-fold (95% CI: 9.5–5.6) higher risk than women in the lowest tertiles.

Carey et al. (10) reported diabetes incidence data from the US Nurses’ Health Study using participant self-reporting mechanisms, validated via a random sample of medical records. They assessed the relative risk for the 90th percentile of WHR (0.86) vs. the 10th percentile (0.70) and the 90th percentile of WC (92 cm) vs. the 10th percentile (67 cm) and concluded that both measures (as well as BMI) were powerful independent predictors of type 2 diabetes in US women.

In particular, Carey et al. (10) argue that WHR and WC are independent predictors of type 2 diabetes throughout the observed range of values. This contrasts with previous studies suggesting that measures of central adiposity might provide additional information on diabetes risk beyond that provided by BMI only in the upper extremes of marginal central obesity distributions.

### Iliac circumference and incidence of diabetes

Chihaoui et al. (11) used IC as the measure of abdominal obesity. A 10-year prospective study of subjects aged ≥ 30 living in Tunis showed that IC is a risk factor for both type 2 diabetes and impaired glucose tolerance, but multivariate analysis indicated it was an independent risk factor for conversion to either condition only in men.

### CT scan assessed abdominal fat and incidence of diabetes

Boyko et al. (12) measured IAFA based on CT scans in second-generation (nisei) and third-generation (sansei) Japanese Americans without diabetes, of whom 22.4% and 5.7%, respectively, developed diabetes, as defined by the American Diabetes Association (13). In both groups, IAFA was a significant predictor of diabetes incidence even after adjustment for BMI, total body fat area and subcutaneous fat area.

### Pooled analysis

We included data on 15 independent cohorts from the 10 included studies in the statistical analysis. All cohorts were adjusted for age, eight were adjusted for BMI and most included a range of other adjustment factors.

**Figure 1:**
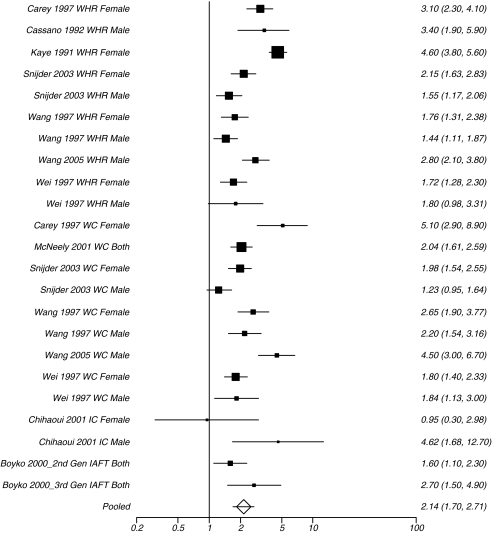
Odds ratios and 95% confidence intervals for incidence of type 2 diabetes

Figure 1 shows that four cohorts had OR > 4.0, two using WC [females (10), males (5)], one using WHR [females (9)] and one using IC [males (11)].

Only one cohort had an OR < 1 [females (11)]. The authors of this paper comment that the large proportion of their sample lost for follow-up (52%) may be a source of bias and may consequently have led to an underestimation of the incidence of type 2 diabetes. Across all the cohorts the pooled OR was 2.14 (95% CI: 1.70–2.71; p < 0.0001) (see Figure 1).

In studies where both WC and WHR were used, the confidence intervals around the ORs for the two measures overlapped. In a meta-regression model, WC was slightly more predictive than other measures used in the studies although this was not statistically significant [ratio of OR 1.11 (95% CI: 0.88–1.39; p = 0.32)].

Follow-up periods ranged from 2 to 18 years. The predictive value of abdominal obesity reduced slightly with follow up, although this was not statistically significant [ratio of OR 0.76 (95% CI: 0.47–1.25; p = 0.24)].

There was no evidence of a gender specific effect (p = 0.28). We examined the potential for publication bias, and found no relationship between the study standard error and the study effect size (p = 0.39).

## Discussion

Our analysis compared the quantitative findings of all available epidemiological studies and shows that abdominal obesity, identified through a variety of measures, significantly raises the risk of type 2 diabetes across a range of different ethnic groups. Although adjustment factors varied, all the cohorts were adjusted for age and eight were adjusted for BMI, which we did not consider to be a measure of abdominal obesity. This gives us added confidence in the overall conclusion that, on average, raised abdominal obesity increases the risk of type 2 diabetes more than twofold.

When we commenced our work there was no comprehensive review examining the relationship between measures of abdominal obesity and the incidence of type 2 diabetes. When our work was completed a review addressing this issue albeit using different methods has been published, finding similar over all results to our own (14). Our study adds independent confirmation of the findings of that study, but in addition provides statistical comparison between WC and other methods of measurement used in the studies, which is not undertaken directly by Vazquez et al. (14).

No heterogeneity in the predictive value of different measures of abdominal obesity was identified. This suggests that WC (the most straightforward measure of abdominal obesity used in the studies) may be sufficient to identify subjects at raised risk. A similar finding has recently been reported in relation to the risk of cardiovascular disease (CVD); Koning et al. (15) found that a 1 cm increase in WC is associated with a 2% increase in the relative risk of future CVD, and the difference between WC and WHR in terms of strength of association is not significant.

Different measures may capture different elements of abdominal obesity. WC cannot distinguish abdominal subcutaneous fat, total abdominal fat and total body fat, and it is strongly correlated with BMI (14), although it performed at least as well as the other measures evaluated here. WC, or more usually maximal abdominal circumference, is easily measured and can be monitored by patients themselves. What this study demonstrates is that whatever measure is used they all show a strong relationship to the incidence of type 2 diabetes. This finding is important because it confirms that clinicians can use a simple measure of abdominal obesity in everyday practice to help identify patients at increased risk of developing type 2 diabetes.

The link between abdominal obesity and diabetes is biologically plausible. Abdominal fat is thought to increase the risk of diabetes through a number of secreted factors including non-esterified fatty acids and adipocytokines including tumour necrosis factor-α and reduced adiponectin. Reduction in WC is associated with an improvement in the circulating levels of these adipose tissue secreted factors. Thus, reducing WC may lead to a lower risk of progression to diabetes, as has been demonstrated in some studies targeting obesity and lifestyle in those at risk of type 2 diabetes (16,17).

As the searches for our review were undertaken, a long-term follow up of multinational monitoring of trends and determinants in cardiovascular disease (MONICA) subjects examining the risk of the development of type 2 diabetes has been published (18). This large study also identified no difference between WC and WHR in predicting risk, and provides further confirmation for our findings.

As a growing array of therapies offers the potential for significant reductions in obesity, effective targeting of these therapies towards those at higher risk and with the most to benefit from treatment may be improved by the systematic measurement of WC alongside other risk factors.
